# Supplementary figures and images for: Structure of the scaffolding protein and portal within the bacteriophage P22 procapsid provides insights into the self-assembly process
Source: PLoS Biol. 2025 Apr 17;23(4):e3003104. doi: 10.1371/journal.pbio.3003104 (PMC12005531; doi:10.1371/journal.pbio.3003104)

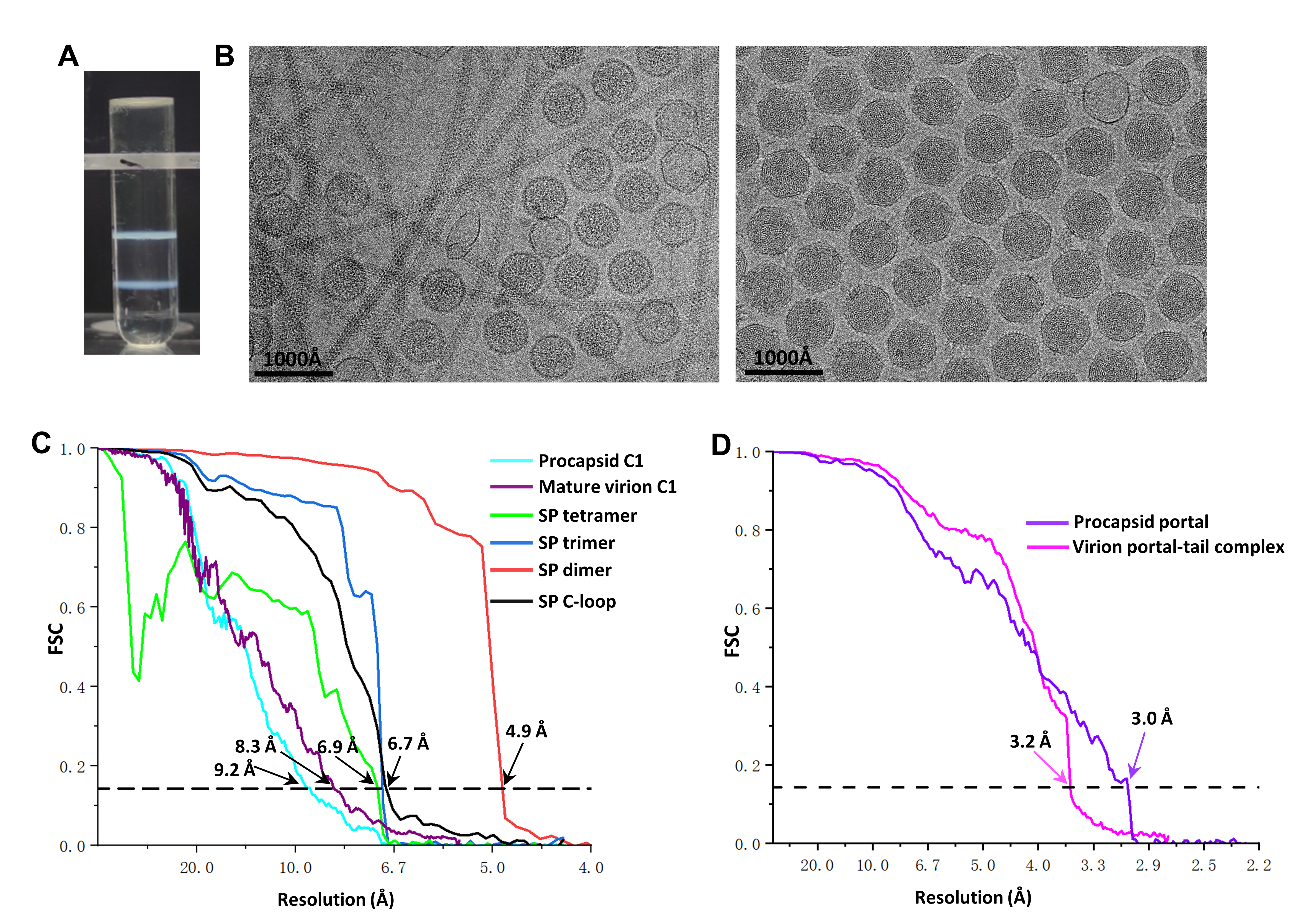

Supplement: S1 Fig — (A) Two bands for phage particles separated through CsCl density gradient centrifugation. (B) Cryo-EM images of P22 particles from the upper (left) and lower (right) bands. (C) Structural resolutions of the overall symmetry-mismatch of the mature virion and procapsid as well as the dimer, trimer, tetramer, and C-loop of the SP in the procapsid were estimated based on Fourier shell correlation (FSC) criteria by using the gold-standard procedure. (D) Structural resolutions of the portal in the P22 procapsid and portal–tail complex in the P22 mature virion. The cryo-EM density maps (EMD-61452, EMD-61453, EMD-61454, EMD-61455, EMD-61456, EMD-61457, EMD-61460, EMD-61461) have been deposited in the EM Data Bank. (TIF) [file pbio.3003104.s001.tif]

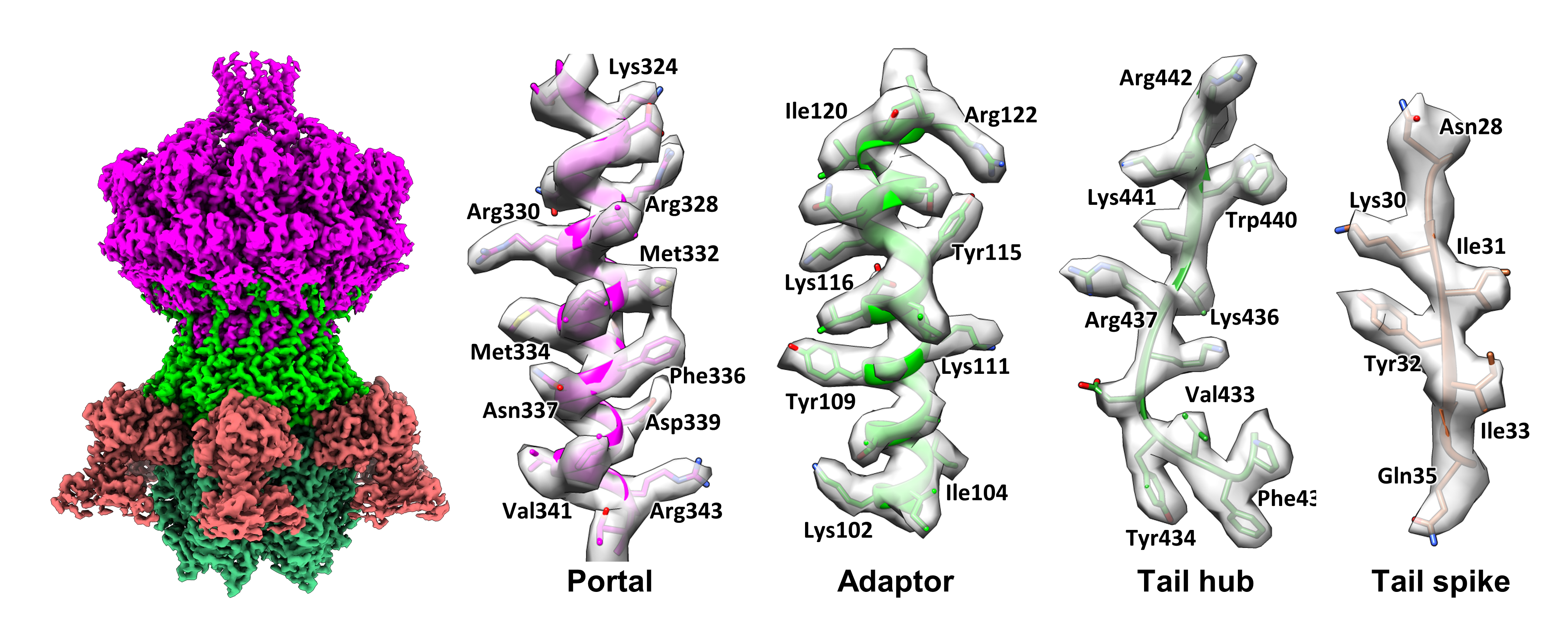

Supplement: S2 Fig — Zoomed-in views of the density maps of the portal, adaptor, tail hub, tail spike in the mature virion (transparent gray) superimposed on models (EMD-61457; PDB ID: 9JG6). (TIF) [file pbio.3003104.s002.tif]

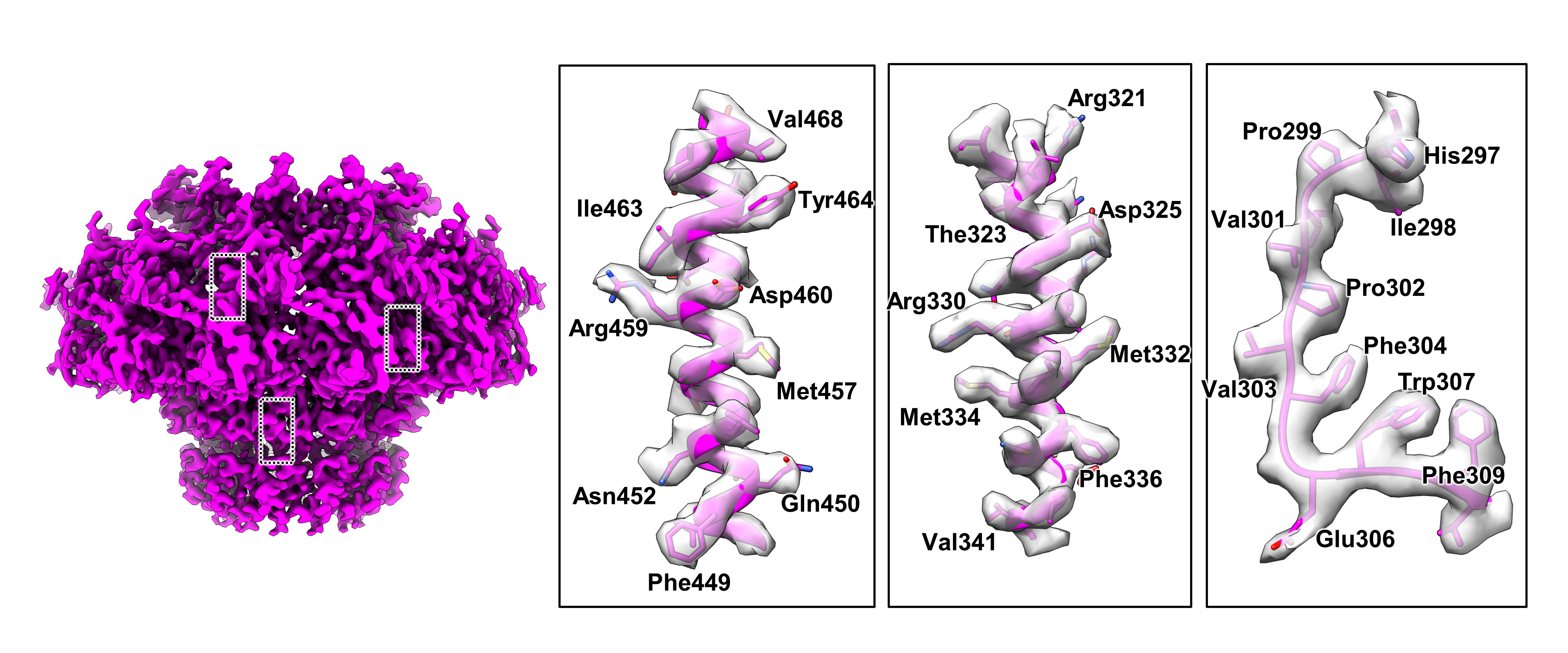

Supplement: S3 Fig — Zoomed-in views of the density maps of three segments (transparent gray) superimposed on models (EMD-61460; PDB ID: 9JGA). (TIF) [file pbio.3003104.s003.tif]

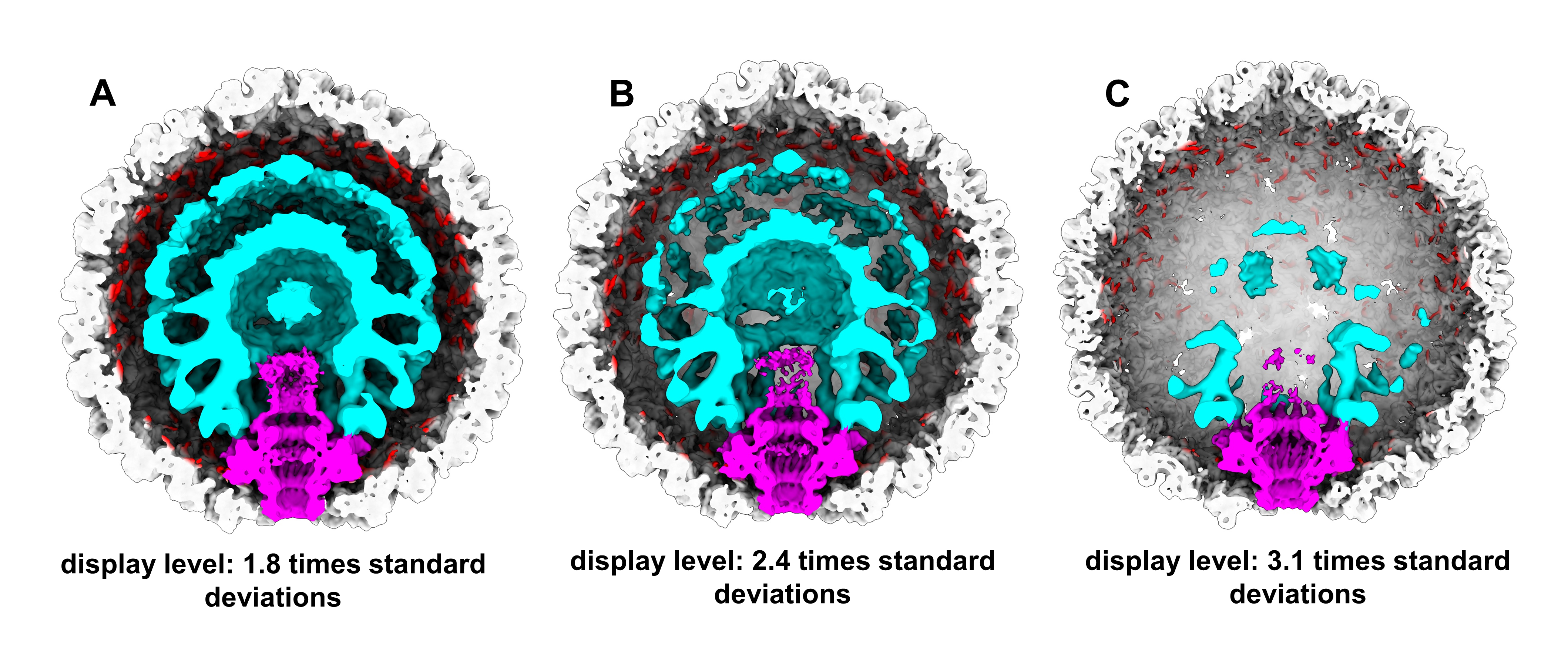

Supplement: S4 Fig — (TIF) [file pbio.3003104.s004.tif]

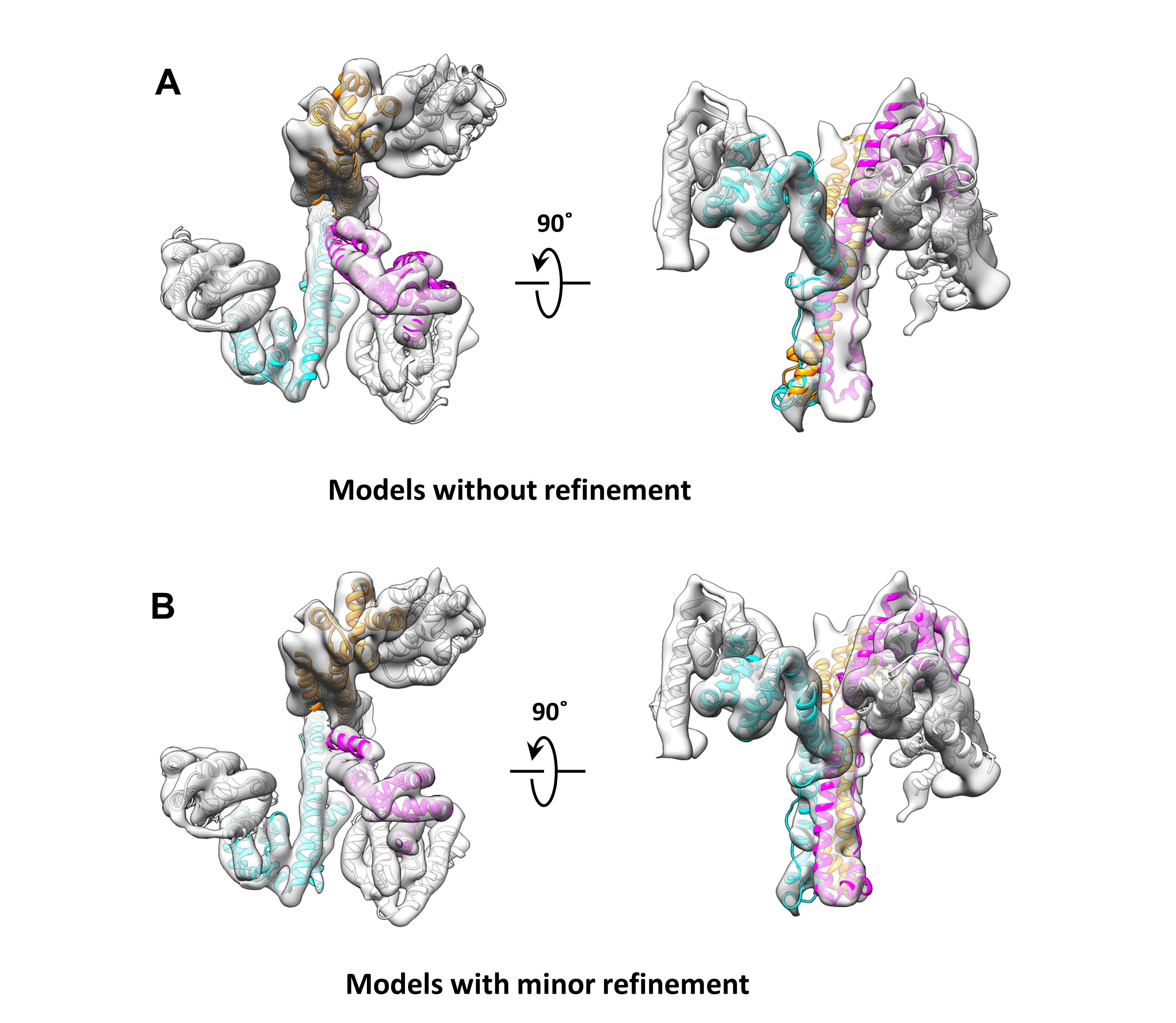

Supplement: S5 Fig — (A) Fitting, without refinement, of three copies of predicted gp8 models (cyan, magenta, and orange) and three base domains (gray) from neighboring gp8 subunits into the density map of the scaffold trimer. The column domains did not fit well into the density map, but the base domains exhibited a good fit. (B) After minor refinement, the predicted gp8 models fit well into the density map of the gp8 trimer (EMD-61455; PDB ID: 9KYY). (TIF) [file pbio.3003104.s005.tif]

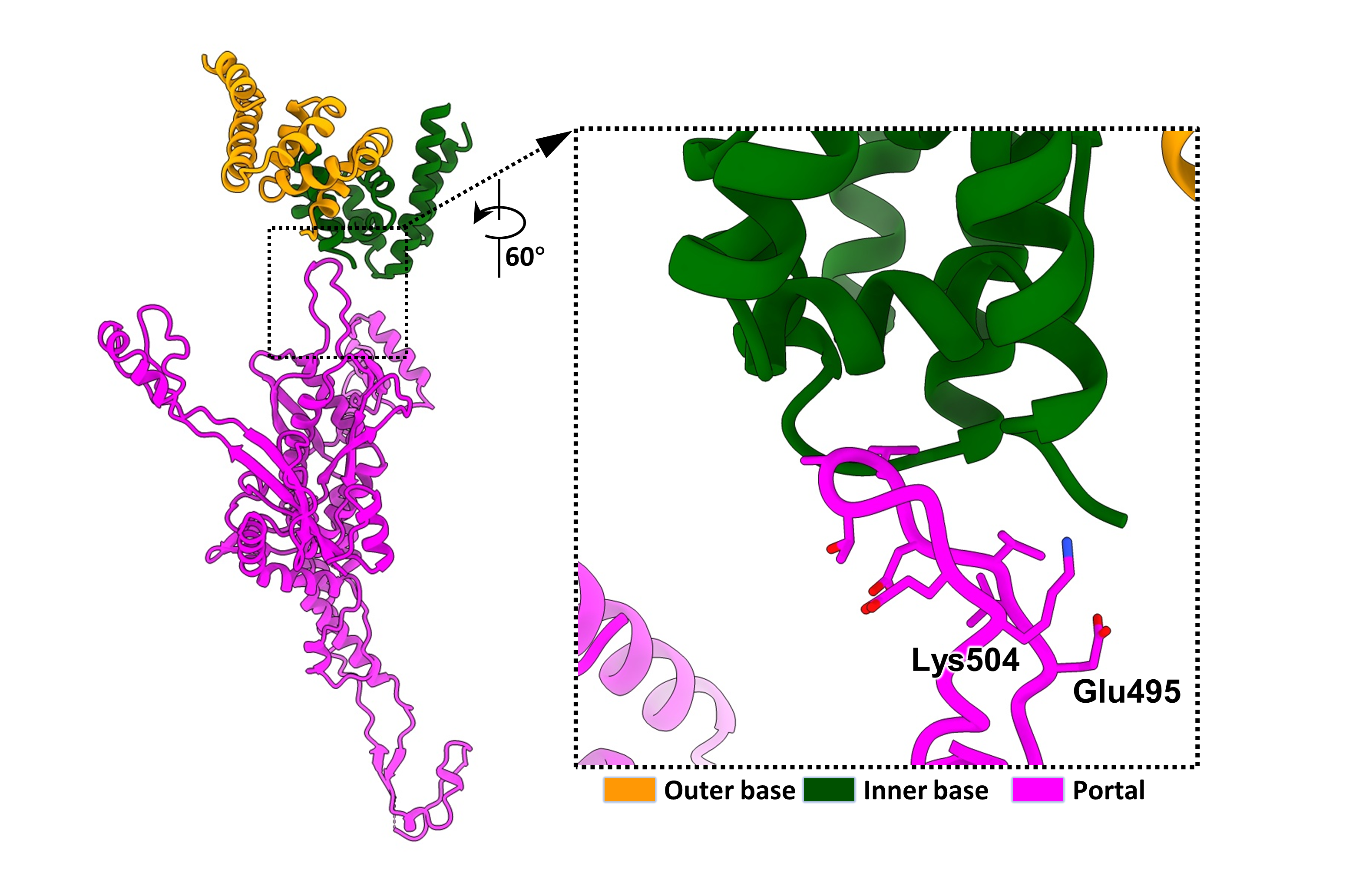

Supplement: S6 Fig — The nodule interacts with the portal through the stacking of the inner base domain on a loop (residues 495–504) of the portal wing (PDB ID: 9KYW). (TIF) [file pbio.3003104.s006.tif]

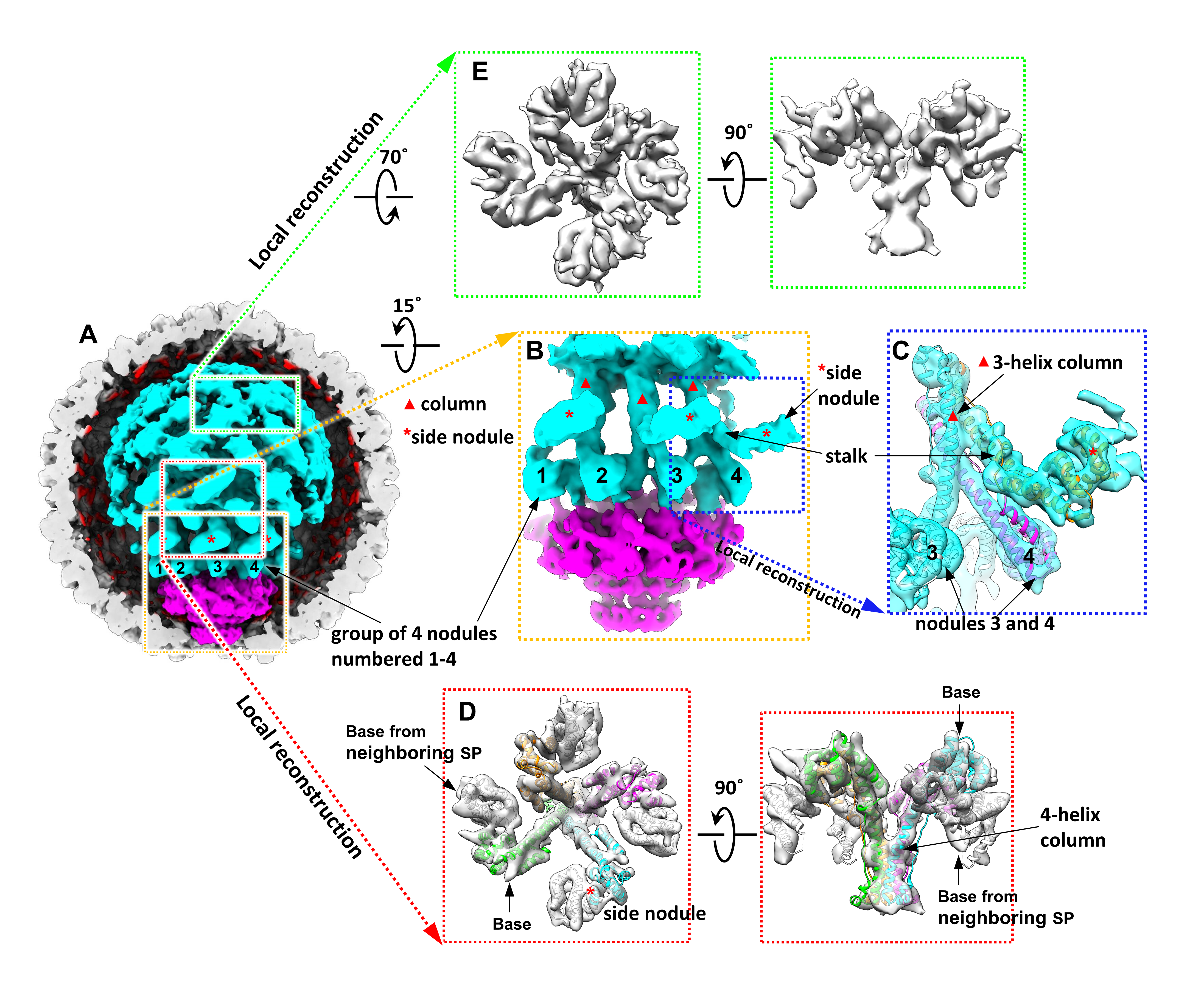

Supplement: S7 Fig — These dimers form the outer dome. (A) Structures of the SP complex (cyan) and portal (magenta) within the procapsid (EMD-61454). The front half of the capsid is not shown. (B) Zoomed-in view of the SP complex within the yellow box in panel A. (C) Zoomed-in view of the SP trimer (EMD-61455; PDB ID: 9KYY) within the blue box in panel B. The middle side nodule that covers the trimer is removed for clarity. (D) Zoomed-in view of the SP tetramer (windmill) (EMD-61456; PDB ID: 9KYX) within the red box in panel A. One of the windmill blades is contributed by a side nodule (asterisked). All blades are formed by dimers of the SP base domain. One of the two bases in each blade is contributed by a neighboring SP subunit. (E) Local reconstruction indicates that the top region of the SP complex is also formed by SP tetramers. (TIF) [file pbio.3003104.s007.tif]

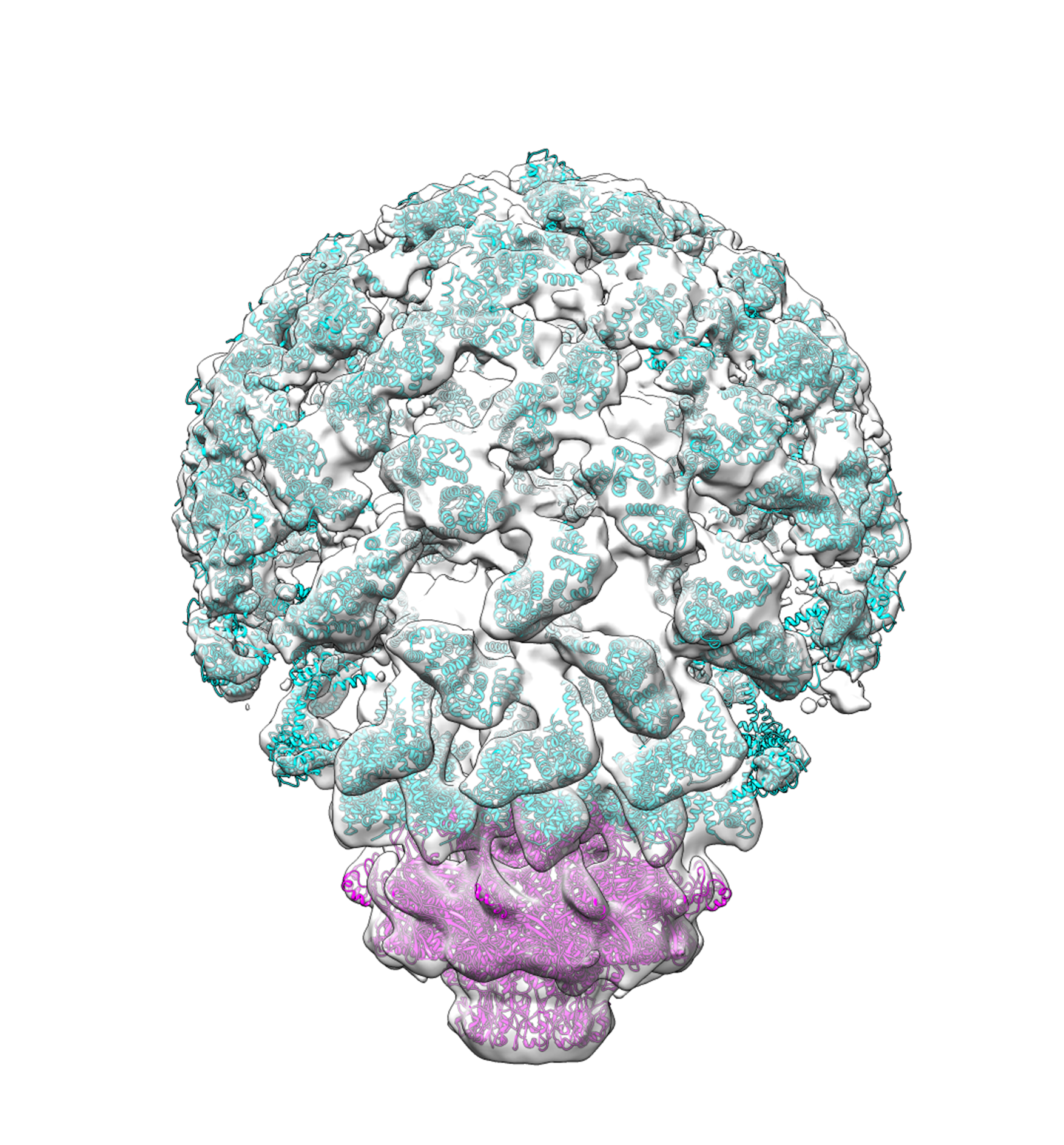

Supplement: S8 Fig — (TIF) [file pbio.3003104.s008.tif]

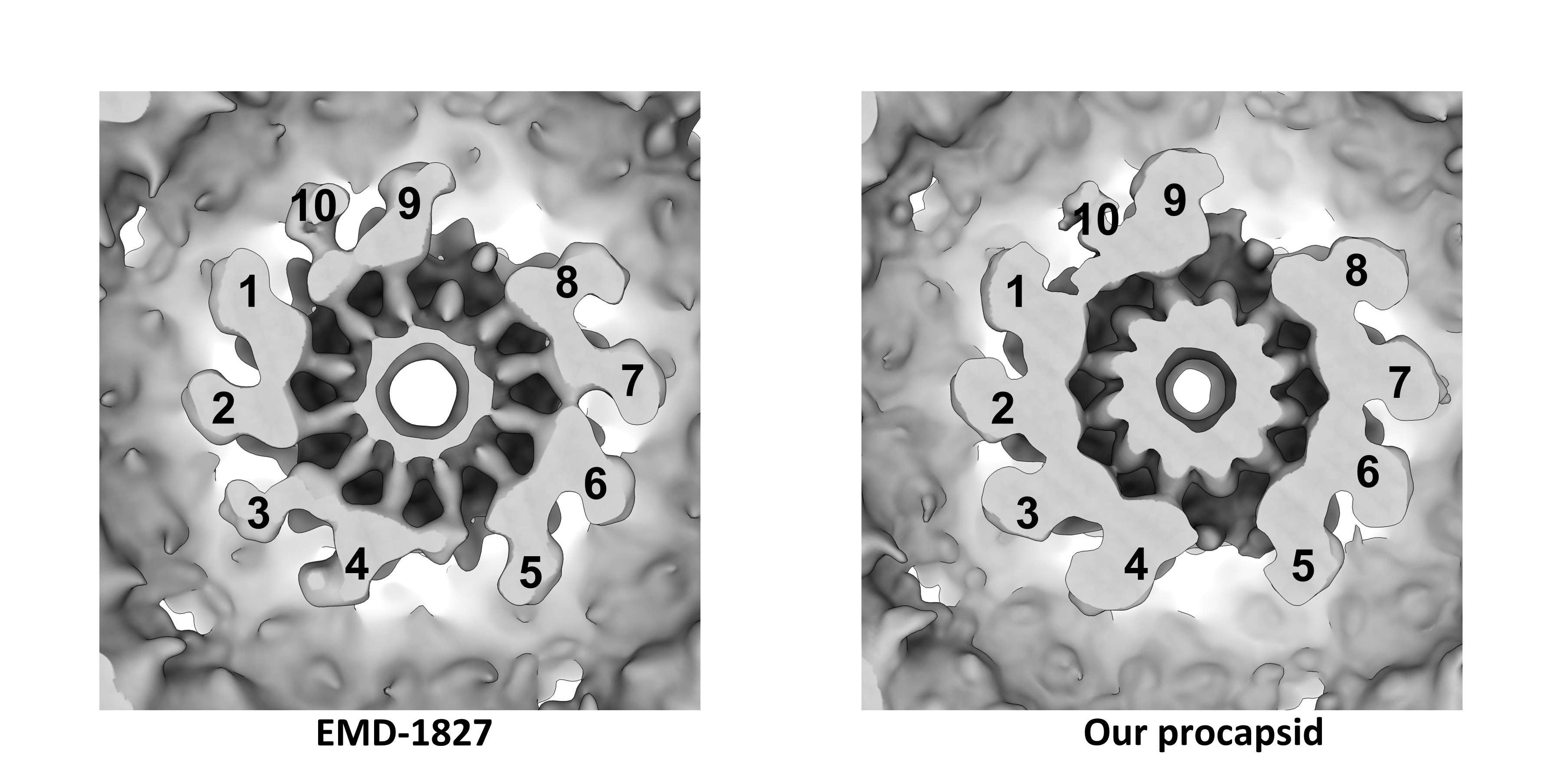

Supplement: S9 Fig — Right: Our structure; the slab-view is identical to that in Fig 2D. Left: EMD-1827, a structure reported by Chen and colleagues in 2011 (PNAS 108:1355–1360). Both structures were filtered to a resolution of 15 Å. (TIF) [file pbio.3003104.s009.tif]

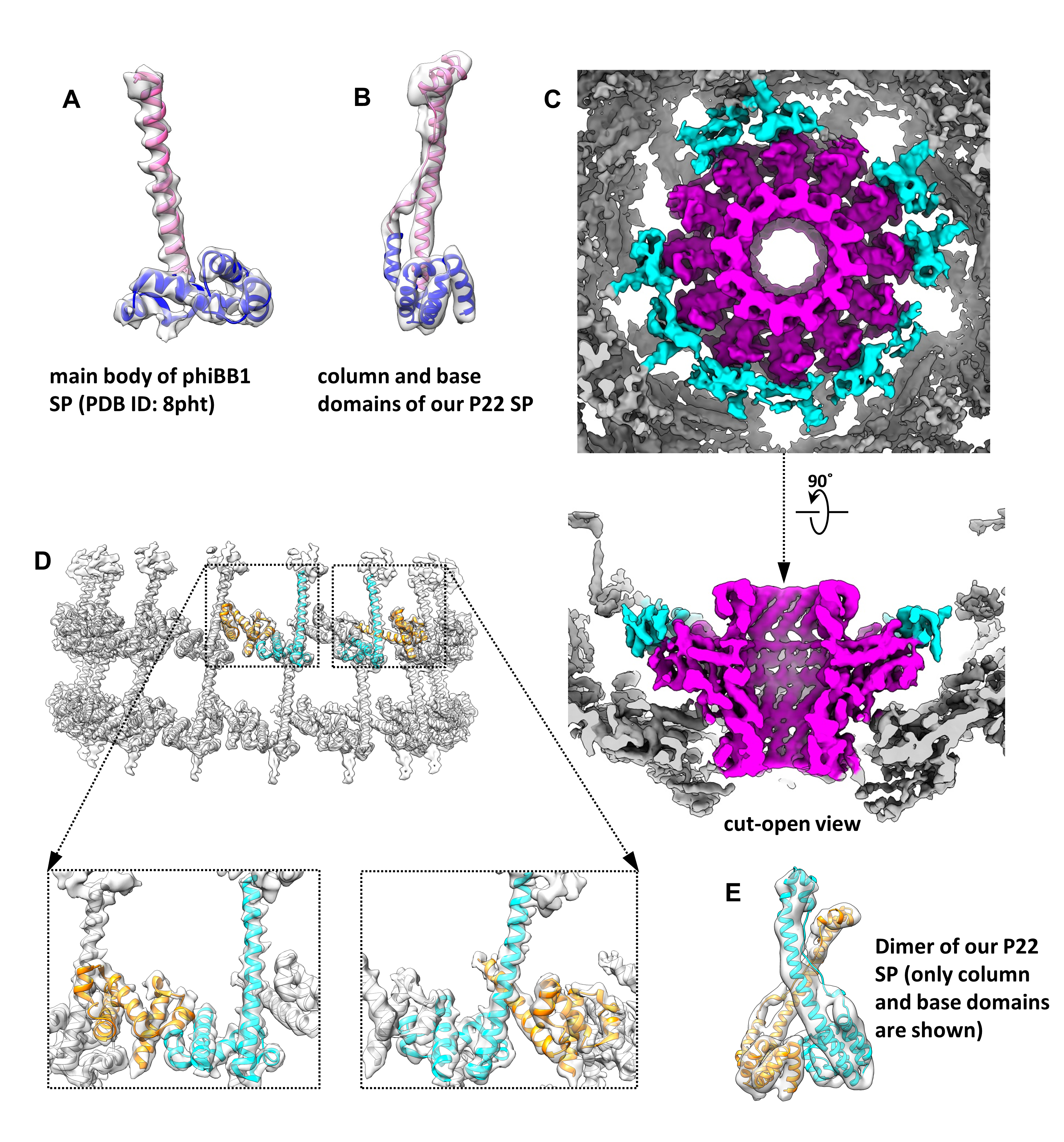

Supplement: S10 Fig — (A) Atomic model of the phiBB1 SP (column and base domains are in pink and blue, respectively) superimposed on a corresponding density map (transparent gray) (PDB ID: 8pht). (B) Column and base domains of our P22 SP subunit. (C) Ten SP subunits (cyan) surround around the portal (magenta) in the phiBB1 procapsid (EMD-17675). (D) Presence of SP subunits beneath the tubular section of the phiBB1 procapsid indicates that SP interactions are mediated by the column and base domains (EMD-17674). SP interactions in phiBB1 are similar to those in P22 (E). (TIF) [file pbio.3003104.s010.tif]
