# Supplementary material for: Structure of the scaffolding protein and portal within the bacteriophage P22 procapsid provides insights into the self-assembly process
Source: PLoS Biol. 2025 Apr 17;23(4):e3003104. doi: 10.1371/journal.pbio.3003104 (PMC12005531; doi:10.1371/journal.pbio.3003104)
Supplement: S1 Table — (PDF) [file pbio.3003104.s011.pdf]

S1 Table. Interactions between the SP hook and portal proteins.

Amino acid in portal subunits	Amino acid in SP subunits
Asp23 (Portal1)	Lys298
Glu24 (Portal2)	Lys278
Asp16 (Portal4)	Lys298
Asp196 (Portal5)	Arg277
Asp43 (Portal5)	Lys286
Glu311 (Portal7)	Lys278
Asp23 (Portal7)	Lys286
Glu24 (Portal9)	Lys278
Asp196 (Portal12)	Lys294
Ser180 (Portal12)	Lys278
Asp43 (Portal12)	Lys286
