# Supplementary material for: Structure of the scaffolding protein and portal within the bacteriophage P22 procapsid provides insights into the self-assembly process
Source: PLoS Biol. 2025 Apr 17;23(4):e3003104. doi: 10.1371/journal.pbio.3003104 (PMC12005531; doi:10.1371/journal.pbio.3003104)
Supplement: S2 Table — (PDF) [file pbio.3003104.s012.pdf]

S2 Table. Refinement and model statistics.

**Data collection**								
Electron microscopy	FEI 300 kV Titan Krios G3i, Gantan K3 camera							
Pixel size (Å)	1.06				1.36			
Defocus range	-1.6 to -2.2μm							
Electron exposure	32 e-/Å2							
Total micrographs	P22 procapsid				P22 mature virion			
	4,668				3,733			
**Local reconstruction**								
	procapsid portal	virion portal-tail complex	procapsid c1	mature virion c1	scaffold dimer	scaffold trimer	scaffold tetramer	scaffold C-loop
Symmetry imposed	C12	C6	C1	C1	C1	C1	C1	C1
Total particles	22,169	82,501	22,169	82,501	199,080	199080	33180	21884
Final particles	19,610	71,020	16,590	71020	16520	141870	21650	17,501
Pixel size (Å)	1.06	1.36	2.12	2.72	1.06	2.12	2.12	2.12
Resolution(Å)	3	3.2	9.2	8.3	4.9	6.9	6.9	6.7
B-factors	80	110	200	180	120	160	160	150
EMDB ID	EMD-61460	EMD-61457	EMD-61454	EMD-61453	EMD-61452	EMD-61455	EMD-61456	EMD-61461
pseudo-atomic model ID	/	/	/	/	9KYV	9KYY	9KYX	9KYW
**Atomic models refinement/statistics (phenix.real_space_refine）**								
	P22 procapsid portal				P22 mature virion portal-tail complex			
PDB ID	9JGA				9JG6			
Initial model used (PDB code)	ab-initio				ab-initio			
Correlation coefficient (model to map fit)	0.8052				0.8645			
**Model composition**								
Number of chains	12				48			
Nonhydrogen atoms	52692				115734			
Residues	6492				14622			
**R.m.s. deviations**								
Bond lengths	0.006				0.003			
Bond angles	0.731				0.6			
**Validation**								
MolProbity score	2.13				1.8			
Clash score	12.28				6.79			
Rotamer outliers (%)	0				0.07			
**Ramachandran plot (%)**								
Favored	90.81				93.46			
Allowed	9.19				6.51			
Outliers	0				0.03			
