# Supplementary material for: Structure of the scaffolding protein and portal within the bacteriophage P22 procapsid provides insights into the self-assembly process
Source: PLoS Biol. 2025 Apr 17;23(4):e3003104. doi: 10.1371/journal.pbio.3003104 (PMC12005531; doi:10.1371/journal.pbio.3003104)
Supplement: Attachment — Submitted filename: responseToReviewers (9).docx [file pbio.3003104.s017.docx]

We would like to thank the reviewers for their critical review of our work and for their constructive suggestions on how to improve the manuscript. The manuscript has been revised to address all comments and suggestions. In particular, we have added Fig. S7, Movies S1 and S2, and Table S1 to the updated manuscript. For ease of reading, we have copied the original comments in black and marked our responses in blue. The page and line numbers provided correspond to those in the revised manuscript with tracked changes (manuscript_changes_tracked.pdf).

Reviewer #1: PBIOLOGY-D-24-02541R1

By Co-authors and Hongrong Liu

The manuscript "Structure of the scaffolding protein and portal within the bacteriophage P22 procapsid provides insights into the mechanism of assembly" by Xiao and co-authors has been submitted to PBIOLOGY.

Phage P22 is a well recognised model object the structure of which has been studied in different aspects for nearly two decades by many different groups. A role of the scaffolding protein (SP) in bacteriophages is rather well known: it is essential for the self-assembly of phages; but, on the other hand, it is still baffling how does SF initiates that process and what would be a sequence of interactions between SF, portal and capsid proteins.

The manuscript reports a structure of the SP of phage P22 at a low resolution within the procapsid and describes possible distribution of the SP within the phage procapsid. The authors describe some suggestions on interactions between SP, the portal complex and capsid proteins of the outer shell of the phage. The authors provide some ideas on a possible sequence of self-assemble process related to other phages.

We thank the reviewer for the positive evaluation of our manuscript and the suggestions for improvement.

However it must be mentioned, that the portal protein itself is generally considered to be an initiator of the portal complex, while the scaffolding protein plays a crucial role in facilitating its proper incorporation into a procapsid and guiding the overall assembly process of the viral capsid, rather than initiating the assembly of the portal complex (Dedeo CL, Cingolani G, Teschke CM. Portal Protein: The Orchestrator of Capsid Assembly for the dsDNA Tailed Bacteriophages and Herpesviruses. Annu Rev Virol. 2019 Sep 29;6(1):141-160. doi: 10.1146/annurev-virology-092818-015819). So, the authors of the submitted MS are not right (lines 67–68 and somewhere else), when writing in the abstract (and in the MS), that "the SP catalyses the formation of the dodecameric portal".

We agree with the reviewer that “the scaffolding protein plays a crucial role in facilitating its proper incorporation into a procapsid and guiding the overall assembly process of the viral capsid”. The scaffolding protein is required both for the incorporation of the portal complex and for the assembly of dodecameric rings from monomers (see page 145 in the paper mentioned by the reviewer: Dedeo et al., Annu Rev Virol. 2019 Sep 29;6(1):141-160). Moreover, previous biochemical data suggested that the scaffolding protein interacts preferentially with portal monomers as opposed to portal rings, lending further evidence to the role of scaffolding protein in the oligomerization of portal rings (see page 145 in the aforementioned paper). The portal monomers alone can polymerize into dodecameric rings but do so less efficiently (Moore SD, Prevelige PE, Jr. Structural transformations accompanying the assembly of bacteriophage P22 portal protein rings in vitro. J Biol Chem. 2001;276(9):6779-88.).

The suggestion that "the SP catalyses the formation of the dodecameric portal" is based on a previous study (Motwani T, et al. A viral scaffolding protein triggers portal ring oligomerization and incorporation during procapsid assembly. Sci Adv. 2017;3(7):e1700423) and our structure of the scaffolding protein and portal complex within the P22 procapsid.

In the revised manuscript, we have changed the word “catalyzes” to “plays a role in” (line 35).

While the topic of this MS is interesting and important, the MS is rather difficult to read, the reasoning of conclusions has to be improved: the authors express certain general ideas, then go to some details, then step back again to a global idea and then return to the details in the figures. Figures should be improved by the proper labelling of details discussed in the MS.

We thank the reviewer for the positive evaluation of our manuscript and for pointing out readability concerns. We chose to move from describing low-resolution to higher-resolution structures in one figure (Fig. 2) and then from low-resolution to higher-resolution structures in the next figure (Fig. 3) due to the sophisticated structure of the portal-scaffold complex, requiring us to use multiple display levels in Chimera software to indicate regions of interest in the low-resolution structure. We then used a local reconstruction method to improve the structural resolutions of regions of interest.

We have divided the section “Scaffolding protein in the capsid” into four sections (lines 176, 195, 246, and 284) and added labels in Figs. 2 and 3 to improve the readability. We have also added Fig S7. to illustrate the organization of the scaffolding protein subunits.

Several structures were analysed and discussed by the authors; however, they did not submit any atomic models to the EMDB data base which would allow to the reviewers to make their assessments, and to find if any possible confusions can be revealed. These "data" were NOT available.

We have uploaded the EMDB and PDB data (page 1, lines 22-25).

The methods part is not written in the required style: if one would like to use similar procedures and/or software, the descriptions given by the authors are very sketchy, and the appropriate references are not given. The authors mentioning the references 57, 58, 59, 60, and 61; however, the software links, descriptions of the algorithms, and principles of the assessment of the reconstruction's quality were not provided in these articles. No references were given related to the methods used by the authors of this MS such as Symmetry-mismatch and local reconstruction (the essence of the text in lines 459-477 is extremely far from to be obvious).  What was an advantage in usage of these homemade programs and which algorithms that were used in the current analysis was not explained. It seems that the authors were not willing to share their expertise and approaches in image analysis with readers. The lines 483-487 have to be clarified in a more explanatory way: how the local reconstruction has been done, on which algorithms it was based, how many images and what symmetry were used.  More details should be given in lines 138-141.

We have now added comprehensive descriptions of our programs, including the algorithms used, the number of images, the symmetry applied, and the specific steps for local reconstruction (see below). Our software package can be downloaded from <https://doi.org/10.5281/zenodo.8378566>. The advantage of our algorithm lies in its efficient use of calculation results and leveraging the fixed spatial relationships between neighboring local structures to obtain initial orientation information. This approach significantly reduces computational resources and time.

We have expanded the paragraph in the method into two paragraphs (lines 540–568):

On the basis of the orientation and center parameters of the procapsid that were obtained through symmetry-mismatch reconstruction, the structures of the portal and SP complexes were locally reconstructed, including the nine nodules (dimers of the SP base) on the portal wing. Reconstruction was conducted at a resolution of 20-Å without imposing symmetry using 22,169 images of procapsid particles. The nine nodule structures located on 12-fold symmetrical wing domains were then averaged. A 3D mask model was generated to iteratively refine the orientation and center of the nodule structures in the procapsid particle images using our local_refine and local_reconstruct software, which were designed according to a model-based approach algorithm [63]. Finally, 199,080 nodule images in the 22,169 images of procapsid particles were selected for reconstruction of the nodule structures at a resolution of 4.9 Å.

Based on nodule orientation and centers, we included the SP trimer in our reconstruction, given that one base in the nodule belong to the SP trimer (Fig. 2E, F, and K). Through iterative local refinement and reconstruction, the structure of the SP trimer was obtained at a resolution of 6.9 Å. The SP tetramer was reconstructed via the same approach. A total of 33,180 SP tetramer images within the 22,169 images of procapsid particles were selected for reconstruction at a resolution of 6.9 Å without imposing symmetry. We selected 21,884 particles for reconstruction of the C-loop structure at a resolution of 6.7 Å. The resolutions of all reconstructions were estimated using Fourier shell correlation (FSC) criteria with a cut-off of 0.143. Our software package can be downloaded from the following website: https://doi.org/10.5281/zenodo.8378566.

Terminology used in the methods for the data collections and image processing was rather unusual: it would be better if the authors will look at other paper and will used more standard terminology (and for the sample preparation as well).

We have revised the data collection and sample preparation paragraphs accordingly (lines 483–504).

A set of terms used for the SF domain designation was confusing and badly explained such as a process of oligomerisation, locations of domains in the oligomers: nodule, GOF, column, blade, hook, long loop, helix, base, windmill. It seems that majority of them are overlapping. Please provide the appropriate protein sequences where domains have to be indicated.

We agree that the nomenclature of the SP complex is complicated, given its sophisticated structure. For clarification, we have added (1) labels for columns, side nodules, and a group of one nodule and two groups of four nodules in Fig. 2C–E; (2) windmill blade labels in Fig. 3A; and (3) labels for the first and last amino acids of the SP (gp8) domains in Fig. 2G.

Each nodule-like structure (including side nodules) is formed by a dimer of the SP base domain (Fig. 2D and H). Each column-like structure is formed by a helix bundle of three SP column domains (Fig. 2E and F), or a helix bundle of four SP column domains (Fig. 3B). Each windmill blade–like structure is formed by a dimer of the SP base domain (Fig. 3B) that is largely identical to the nodule. We have added Fig. S7 to describe these structures within a single figure.

It should be written in Methods how the authors identified which band corresponds to the fully assembled phages and which one corresponds to the procapsids (confusing lines 136-137). Info on how these bands were identified should be provided.

We identified the upper band as procapsids and the lower band as mature particles using negative-stain electron microscopy (see the following figure). The results were further confirmed through subsequent cryo-EM analysis.

The representative micrographs must be shown at the same magnification (scale bar must be of the same size!), so a reader will be able to identify which are the procapsids and which ones correspond to the fully assembled phages.

We have modified the scale bars in Fig. S1B.

Sizes of particles must be indicated in the text or shown in figures. According to the figures the scale bars given in the images are not correct.

We have added a scale bar in Fig. 1A and C and modified the scale bars in Fig. S1B.

Figures should be improved: partly simplified and better labelled.

We have added (1) labels for columns, side nodules, and a group of one nodule and two groups of four nodules in Fig. 2C–E; (2) labels for the windmill blades in Fig. 3A; and (3) labels for the first and last amino acids of the SP (gp8) domains in Fig. 2G.

There are many minor comments on this MS.  Below is only a part of them:

Line 29. Abstract. Bacteriophages do not include herpesviruses. Rephrase the sentence.

We have modified the indicated sentence.

Line 37, The structures shown in the figures were not at "sub-nanometer resolution".

We have changed the phrase “sub-nanometer resolution” to “at resolutions of 3–9 Å” (see Fig. S1C and D).

Line 88. What is a homology between SF of the Herpesviruses and P22, where is the pocket located if SP is featureless?

The HCMV (Herpesviridae) has two scaffolding proteins (pPR and pAP). The sequence identities between the two HCMV scaffolding proteins and P22 gp8 were 6.8% and 9.6%, respectively. The overall structure of the HCMV scaffold was featureless except for a short α-helix observed in the hydrophobic pocket of the portal crown domain (see Li Z, et. al., Cryo-electron microscopy structures of capsids and in situ portals of DNA-devoid capsids of human cytomegalovirus. Nat Commun. 2023;14(1):2025).

Line 107. "a unique vertex of the coat " -> do the authors have in their mind a mature capsid of P22? P22 does not have any coat compared to the herpes viruses that have lipid coats.

P22 does not have a lipid coat. We have changed the word “coat” to “capsid” (line 109).

Line 108, When the ejection proteins are delivered into the phage capsid? How?

According to the organization of the ejection proteins and SP observed in our study, we speculate that the ejection proteins were co-assembled with the SP during procapsid assembly.

Lines 119-122. This text is related to methods and should be explained there with more details and assessments of criteria used to make the appropriated decisions during refinements.

We have deleted the sentence “the structure was obtained using cryo-EM single-particle, symmetry-mismatch, and local reconstruction methods” and expanded the description of our methodology (lines 540–568).

Lines 156-168. The proper references related to other structures of the P22 connector have to be provided (EMDB numbers and references to the publications), more clear description of the differences between procapsid and mature capsid should be given. Please try to quantify them, indicate differences (shifts and rotations) in the figures.

We have provided the EMDB number of the P22 procapsid portal structure that was previously published (line 162).

We have also revised the description of the differences between the procapsid and mature capsid and added Movie S1 displaying changes to the portal structure (lines 168–175).

Structural comparison between the portals in the procapsid and mature virion revealed major conformational changes (Fig. 1G and S1 Movie): (i) the top region of the wing domain, which interacts with the SP complex (described below); (ii) the stem and clip domains, which may interact with the terminase complex motor during DNA packaging; (iii) the helical barrel domain, which was not present in the procapsid.

Line 191-194. Explain in a more transparent way why do you talk of the groups of four (GOF - is a rather confusing term, are you implying on the detective story?) if the SP can make dimers, trimers and why tetrameric oligomers are more important? How in a tetramer of SF (GOF) may have three columns? The arithmetic does not work!

We referred to groups of four (GOF) nodules because the SP complex interacts with the portal through GOF nodules. We used the abbreviation “GOF” based on the nomenclature of groups of nine (GON) hexons in adenovirus capsids. For clearer description of the trimers and tetramers in the SP complex, we have inserted Fig. S7 and Movie S2.

***S7 Fig. The SP complex is formed by SP trimers and tetramers, which interact with each other through the formation of SP base dimers. These dimers form the outer dome. (A)*** *Structures of the SP complex (cyan) and portal (magenta) within the procapsid. The front half of the capsid is not shown.* ***(B)*** *Zoom-in view of the SP complex in the yellow box in panel A.* ***(C)*** *Zoom-in view of the blue box in panel B shows a SP trimer. The middle side nodule that covers the trimer is removed for clarity.* ***(D)*** *Zoom-in view of the red box in panel A shows a SP tetramer (windmill). One of the windmill blades is contributed by a side nodule (asterisked). All blades are formed by dimers of the SP base domain. One of the two bases in each blade is contributed by a neighboring SP unit.* ***(E)*** *Local reconstruction shows that the top region of the SP complex is also formed by SP tetramers.*

Line 192 "…there are three columns, each of which is located between the two neighboring nodules …?  It is extremely confusing, the figure does not help to resolve this confusion.

We thank the reviewer for pointing out this problem. We have modified the indicated text as follows: “Three column-like structures, each interacting with two neighboring nodules, were observed on the GOF nodules (Fig. 2E)”. We have also labeled the column-like structures in Fig. 2E. Higher resolution analysis (described further in the manuscript) indicated that each column is a three-helix bundle and each nodule (or side nodule) is a dimer of the SP base domain. Two helices in the three-helix bundle are contributed by two SP subunits, each of which has a base domain contributing to one of the neighboring nodules (see Fig. 2K), and one helix in the three-helix bundle is contributed by a SP subunit, which has a base domain contributing to a side nodule.

Line 193 "..From each column, another nodule extends into the external space" (where is this "space"?) Where is "…a stalk-like structure…" , nothing was properly labelled in the figures.

We have modified the indicated text as follows: “From each column, another nodule extends outward (referred to as a side nodule) through a stalk-like structure.” We have also added stalk, column, and side nodule labels in Fig. 2A, C–F, and K.

Lines 196-201. The text needs more appropriate language, a schematic figure, and improvement in English. Where are the hook, long loop, and helix? Give the sequences and label these domains in appropriate amino acids as in AA length.

We have modified the indicated text as follows: “We applied the local reconstruction on the nodules and columns to improve the structures to resolutions of approximately 8 Å. The nine nodules, which are essentially identical, are formed by α-helices (Fig. 2H and 2J), and all three columns are formed by a three-helix bundle (Fig. 2F). We speculated that these nodules and columns are formed by the SP gp8. Therefore, the structure of gp8 was predicted using AlphaFold2. The predicted gp8 structure consists of a long loop at the N-terminus, followed by a helical region, another long loop and a U-shaped hook (helix-loop-helix motif) at the C-terminus (Fig. 2G). We divided the structure of gp8 into five domains (Fig. 2G): N-loop (residues 1–57), base (residues 58–84 and 163–245), column (residues 85–162), C-loop (residues 246–267), and hook (residues 268–303)” (lines 207–219).

We have now labeled these domains in appropriate amino acids in Fig. 2G.

Lines 223-225. Why the trimers interact with the "neighbouring portal subunits"? Do the trimers of the SF are formed only in the vicinity of the portal complex? Why?

As indicated in Fig. 2K (schematic of panel E), the column (marked with a red triangle) is formed by three copies of the SP column domain, each of which is associated with a base domain. The three SP subunits interact through the three-helix bundle are considered as a SP trimer (see S2 Movie). The SP trimers are formed only in the vicinity of the portal complex, whereas the SP subunits in the remaining part of the SP complex present as tetramer (windmill-like structure).

Notably, in the SP complex, all base domains present as dimers, which form nodules, side nodules, and windmill blades. In Fig. 2K, all base domains form nodules (marked with number 1–4) and side nodules (marked with red asterisks). Thus, the trimers interact with the neighboring portal subunits through nodule-portal interactions (Figs. 2K and S6).

Lines 226-241. Does the GOF (four SFs or not?) differ from the windmill?  How the structure of the "outer dome" has been obtained?  It is confusing: "One blade is a contribution from the side nodule".  (??) Figures do not explain much. And now "We speculated that each blade in the windmill-like structure is formed by two base domains, which is similar to the structure of the nodules." It is difficult to follow. One base is supposed to be from one SP? Or the authors had something else in their mind?  Should it be that a windmill is equivalent to the GOF? Or it consists of the eight SPs?  The authors have to do a better job to explain their ideas.

The “GOF” in the manuscript refers to a group of four nodules. We have added S7 Fig and S2 Movie to illustrate the structures of the four-blade windmill, GOF nodules, side nodules, SP trimers, and tetramers in the SP complex. Each SP molecule has one base domain. The SP complex is formed by SP trimers and tetramers. These trimers and tetramers interact with each other through the formation of the SP base dimers. These dimers form the outer dome. The SP trimers are located around the portal crown, and the SP tetramers are located on the trimers. All blades are formed by dimers of the SP base domain. The outermost part of each blade is not contributed by the gp8 tetramer but contributed by the base domain of a neighboring gp8 subunit (see S7 Fig and S2 Movie).

The sentence on lines 243-247 should be clearer: it is rather confusing in sense of the locations of the SP domains. English must be improved; the explanation should be better.

We have added S7 Fig and S2 Movie to illustrate the SP trimers and tetramers in the outer dome and modified the indicated sentence as follows: “These local reconstructions indicate that the nodules, side nodules, and windmill blades are the dimers of the SP base domains. The column-like structures between the outer and inner domes are the helix bundles, which are formed by each three or four α-helices of the SP column domain (Figs. 2F, 3B, and S7; S2 Movie)”.

In summary, the SP complex is composed of SP trimers and tetramers, which are formed by SP subunits through the formation of three-helix bundle and four-helix bundle, respectively (S2 Movie and S7 Fig). These trimers and tetramers interact with each other through the SP base domains (base dimer), forming a lattice of base dimers in the outer dome (S8 Fig).

Lines 259-268. A role and locations of hooks of the SP should be indicated more accurately. The lines are very confusing. Interactions between SF and portal protein have to be somehow justified and better explained.

We have added the following description of the interactions between the hook, coat protein, and portal protein.

“Our previous icosahedral analysis of the P22 procapsid [21] indicated that each hook interacts with the inner capsid primarily through electrostatic interactions, which are mediated by Arg293 and Lys296 in the hook and by Asp14, Glu15, and Glu18 in the N-arm domain of the coat protein gp5. Superimposition of the portal and hook models onto the asymmetric reconstruction of the procapsid revealed that the asymmetrical interactions between the hooks and portal are likely also electrostatic (S1 Table)”.

Discussion should be much more coherent.  It raises nearly the same questions as the results part: there is no clear explanations of comparison with other phages  and how it can be related to the function of the SP.

Line 292. Why should the SP increase the stability of the portal complex? It is stable even without SFs.

We have deleted the text “…which may increase the stability of the portal” (line 330).

Line 298 . The nucleation of the capsid is the portal complex, the SP is essential for assembly the icosahedral procapsid.

We have modified the sentence to “We propose that the SP and portal protein comprise a scaffold–portal nucleation complex, which is essential for assembly of the icosahedral procapsid.” (lines 335–338).

Lines 303-305. Should be justified and explained in a better way.

We have deleted the sentence “Some coat subunits do not interact with the hooks; these subunits may be secured in place through intrinsic contact with the initially anchored coat subunits” (lines 340–342).

Lines 307-311. Should be better explained using results reported in the references.

We have modified the indicated text as follows: “The inner dome may be formed by the three ejection proteins gp7, gp16, and gp20 because (i) the SP may be required for the ejection proteins gp16 and gp20 to be assembled into the procapsid [19, 39] and addition of physiological quantities of gp16 resulted in an increased rate of procapsid assembly [40]; (ii) the three ejection proteins may interact with each other [41]; and (iii) the inner dome is located around the portal helical barrel domain (although mostly disordered) in the procapsid (Fig. 2B), a place where the three ejection proteins are located in the P22 mature virion [41]” (lines 344–351). We have also inserted the reference [39] (line 346).

Conclusions in the discussion should be more clearly formulated and possibly indicated as i, ii, iii and so on. Lines 341-405 do not indicate that some new information was gained during this study compared to the previously published results by the same and other authors in the previous years.

Of course, it is highly possible that the authors did not describe their achievements in more coherent and well-defined way.

 The structural comparisons with other phages and herpesviruses allow us to summarize the structural similarity between the procapsids of these dsDNA viruses. We have added the following conclusions regarding the SP complex to the manuscript.

“Based on the structure of the portal and SP complex within the P22 procapsid and on structural comparisons with other dsDNA viruses, we conclude that (i) the main body of SP consists of a nodule-like base domain and a long-helix column domain; (ii) within the procapsid shell, the SP subunits interact with each other through the formation of 2-, 3-, and 4-helix bundles with the long-helix column domains, and through the dimerization of the base domain, forming a spherical disordered layer (or layers), which serves as scaffolds for the assembly of the major capsid protein; and (iii) the SP complex interacts with the portal through the base domains, and the structures adjacent to the portal are relatively more ordered than those distal from the portal”.

Reviewer #2:

In this manuscript, the authors present the subnanometer resolution structure of the SP and portal in the procapsid of bacteriophage P22. With the AlphaFold2-predicted SP models the authors show that the SP forms trimers and tetramers that assemble into a dome-like structure. Two SP domains are involved in multimerization, with each trimer interacting with two neighboring portal subunits. The SP also features a loop-hook structure that interacts with the coat protein, aiding in its recruitment during assembly. The loops of the SP subunits are positioned in clefts between adjacent portal subunits. The authors suggest that the conformational changes in the portal during maturation likely trigger SP disassembly and release.

The innovation of this manuscript lies in the authors' careful and sophisticated data processing strategies, which enabled the local refinement of SP proteins despite their potentially flexible arrangement. The map and model show strong agreement at subnanometer resolution, and the introduction is clear and well-referenced. However, the presentation of the results could be improved by considering the following suggestions.

  We thank the reviewer for the positive evaluation of our manuscript and the suggestions for improvement.

Major points:

Related to Figure 1, a supplement movie showing the morphing between the model of the portal in the procapsid and mature virion will be really helpful.

We have added S1 Movie, which displays the conformational change of the portal from procapsid to mature state (line 172).

Figure 2 is overly complex, which may make it difficult for readers to interpret. The current color choices could lead to confusion, as the same colors represent different structures across panels. For example, in Panel G, yellow and purple indicate the C-loop and column of gp8, but in Panel I, yellow represents the outer base while purple means the portal. I suggest selecting distinct colors for the gp8 domains in Panel G to avoid this confusion and improve clarity. Additionally, a movie that gradually zooms in and out to highlight the relative positions of the structures in Figure 2 would greatly enhance clarity. As it stands, understanding the spatial relationships and orientations of the different panels is quite challenging from the static figure alone.

We have changed the color scheme in Fig. 2G–2J and added S2 movie to highlight the relative positions of the trimers and tetramers.

The authors mention that it is unclear why only 9 nodules are present on the dodecameric portal. Could it be that the binding of 8 nodules on one side induces a conformational change at the interface, preventing the binding of all the remaining 4 nodules on the other side? If so, could the authors analyze the portal's asymmetry upon SP binding and include a supplementary figure to illustrate this?

As described in the manuscript, the loop (residues 495–504) of the portal wing domain serves as the interface for interaction with the nodule. It was indeed the case that the structural comparison of the portal subunits that do versus do not interact with a nodule indicated the conformational change of a region (residues 230–271) adjacent to the loop (see the following figure). However, we are unable to conclude whether the conformational change prevented the binding of the nodule or resulted from the absence of the nodule.

*Structural comparison between portal subunits that interact (left) and do not interact (right) with a nodule, indicating a conformational change of a region (approximately residues 230–271).*

Line 246: "approximately 100 models…". Why approximately? How many models are used to generate Fig. S7?

We fitted 96 models of the base dimer into the SP complex density map. We used the phrase “approximately 100 models…” because the densities at the top of the SP complex were too weak to be accurately fitted. We have revised this text to “In total, 96 models of the base dimers could be fitted to the overall density map of the outer dome (S8 Fig)” (lines 274–275).

Minor points:

Line300: what does five hexons mean here since authors provides evidence from literature that coat subunits do not form pentamers and hexamers before assembling into procapsid.

We have changed the word “hexons” to “hexamers” (lines 289 and 339). The major capsid protein only forms hexamers and pentamers after assembling into the procapsid (or capsid).

Line 426: To check purity, did the authors run an SDS gel or using negative staining EM?

We identified the upper band as procapsids and the lower band as mature particles using negative-stain electron microscopy (see the following figure). The results were further confirmed through subsequent cryo-EM analysis.

In Fig. S1B In the image for the upper band of the procapsid, what is the filament-like structure in the micrographs.

The filament-like structures are bacterial flagella.

S1 Table: symmetry is mispelled and was the dataset for procasid portal used from a previous study (https://www.mdpi.com/1999-4915/15/2/355 supplementary table 1)

We have corrected the misspelling. We used the same dataset for the structural analysis of the portal and SP complex.

Reviewer #3 (Xinghong Dai, signs review):

In this article, Hao Xiao et al report the structural studies of phage P22 procapsid, with a focus on the scaffolding protein (SP), and the insights into capsid assembly mechanisms of dsDNA viruses. The successful determination of the SP structure for any spherical phage at secondary-structure resolution represents a great triumph by the symmetry mismatch method, which is pioneered by the senior authors of this paper. The structures are beautifully solved and presented, and the insights brought by these structures are enlightening. I believe this paper would be of great interest to a broad spectrum of readers.

 We thank the reviewer for the positive evaluation of our manuscript and the suggestions for improvement.

While this paper is very well written and I enjoyed the reading a lot, here are some suggestions for minor revisions and questions:

1. It would be great if Fig. 1G can be companied with a simple movie morphing from the immature portal to the matured one, preferably showing both the individual subunits and the dodecamers.

We have added S1 Movie displaying the conformational change of portal from procapsid to mature state.

2. The geometry in Fig. 2K is very complicated. It is better to differentiate each subunit of gp8 with different color so that it is easier to understand!

We have added S7 Fig and S2 Movie to illustrate the structures of the four-blade windmill, GOF nodules, side nodules, SP trimers, and tetramers in the SP complex.

3. Where on the coat protein does the SP hook domain bind? This should be described, preferably with an extra panel in Fig. 4. Is it possible that one SP hook can bind to a coat protein dimer (e.g., if the binding position is across a coat dimer interface)? This seems tempting as 200 SPs vs 415 coat proteins is roughly 1:2.

We analyzed the interaction between the SP hook and coat protein at near-atomic resolution in our previously published paper (Hao Xiao, et. al. Assembly and capsid expansion mechanism of bacteriophage P22 revealed by high-resolution Cryo-EM structures viruses, 2023, Vol. 15, 335, Figure 2). We have added a description and a reference to this paper in the revised manuscript (lines 296–302). All hooks have a specific interaction with the coat protein and the binding position was not located across the coat dimer interface.

4. Is it possible that the position #10 of the SP nodule on the portal, which has very weak densities compared to the others, and the two empty positions, are occupied/hindered by the ejection proteins?

We ascribed the weak densities of the nodule to the flexibility of the nodule on the portal. We did not find any evidence that the position #10 and the empty positions were occupied or hindered by the ejection proteins.
