# Supplementary material for: Structure of the scaffolding protein and portal within the bacteriophage P22 procapsid provides insights into the self-assembly process
Source: PLoS Biol. 2025 Apr 17;23(4):e3003104. doi: 10.1371/journal.pbio.3003104 (PMC12005531; doi:10.1371/journal.pbio.3003104)
Supplement: Attachment — Submitted filename: responseToReviewer.docx [file pbio.3003104.s018.docx]

Reviewer #1: Review: PBIOLOGY-D-24-02541R1

The manuscript (MS) PBIOLOGY-D-24-02541R1 reports on the study of interactions between the scaffolding protein (SP) and the portal complex in phage P22, an assessment of possible distribution of the SP within the phage procapsid using structural analysis by cryoEM. The authors have obtained a few structures at a range of resolutions between 4-8 A. The authors make suggestions which interactions could take place between the SP and the phage portal complex. The topic of this MS is interesting and could be important.

The authors improved the MS, it has now better logistic and is easier to read. However, there are still some issues that make appreciation of the results obtained rather difficult (these issues have been mentioned before and indicated by the second reviewer as well).
 We thank the reviewer again for the positive evaluation of our manuscript and the following suggestions for improvement.

The authors were still not willing to share with readers details related to the image analysis of the asymmetrical elements within the large bio-complexes. Unfortunately, the refences on image processing (given now) do not provide the succinct information as well. How a resolution of the maps obtained was assessed? Where are the FSC curves? How good was the fitting quality of the predicted structures assessed?
 We have added a more detailed descriptions of our programs for symmetry-mismatch and local reconstruction (lines 614-677).

The structural resolutions of the overall symmetry-mismatch of the mature virion and the procapsid as well as the dimer, trimer, tetramer, and C-loop of the SP in the procapsid were estimated based on Fourier shell correlation (FSC) criterion using gold-standard procedure. We have revised the figure legend of Fig. S1C accordingly. The FSC curves are in Fig. S1C.

The correlation coefficient (CC) between the gp8 density map and the predicted scaffolding protein gp8 model is 0.737, and the CC between the gp8 density map and the density map-constrained refined model of gp8 is 0.847.

It is interesting, that the authors were concentrated exclusively on interactions of the SP with the portal complex and completely omitting consideration of the SP interactions with the capsid proteins (CP). The SPs induce the CPs oligomerisation, help to form hexameric or pentameric CP oligomers, and then combine them to form the procapsids, this process is accompanied by interactions with other accessory phage proteins. It remains unclear which kind of oligomers form the SPs themselves. It would be useful to have some hypothesis that would explain a mechanism of the self-assembly of procapsids based on the SP activity.
 We have revised the hypothesis that would explain a mechanism of the self-assembly of procapsids based on the SP activity according to the reviewer’s suggestion (lines 448-450).

Previous biochemical results indicated that P22 SP forms a mixture of monomers, dimers, and tetramers in solution (see ref. 46). These results are consistent with the organization of the SP complex, in which the SP subunits form dimer (through base domain) and tetramer (through column domain). Therefore, the oligomers of the SP may be dimer and tetramer (see lines 457-460).

There are many small details that have to be still corrected, mainly of a linguistical sort such as: repeated expressions, strange sentences etc. Somebody has to check the English of the MS and remove unnecessary repeats of words and expressions. The English of some sentences must be corrected.
 We had our manuscript revised by a professional editing service to improve the quality of the writing and language (The changes were tracked in Manuscript-tracked.pdf).
